# Hemorrhagic PitNets Are Associated with Previous Vascular Events and Result in Worse Endocrine Outcome

**DOI:** 10.3390/cancers16234105

**Published:** 2024-12-08

**Authors:** Harold F. Hounchonou, Josef M. Lang, Katja Döring, Christoph Terkamp, Holger Leitolf, Shadi Al-Afif, Elvis J. Hermann, Christian Hartmann, Joachim K. Krauss

**Affiliations:** 1 Department of Neurosurgery, Hannover Medical School, 30625 Hannover, Germany; 2 Institute for Diagnostic and Interventional Neuroradiology, Hannover Medical School, 30625 Hannover, Germany; 3 Department of Gastroenterology, Hepatology, Infectiology and Endocrinology, Hannover Medical School, 30625 Hannover, Germany; 4 Department of Neuropathology, Hannover Medical School, 30625 Hannover, Germany

**Keywords:** pituitary neuroendocrine tumor, pituitary apoplexy, intratumoral hemorrhage, transnasal-transsphenoidal approach

## Abstract

Background: Pituitary apoplexy is a potentially life-threatening condition that most often results from hemorrhage into a preexisting pituitary neuroendocrine tumor (PitNet) presenting with acute headache, visual impairment and endocrine dysfunction. Here, we aimed to identify factors associated with hemorrhage and present the pituitary hormonal status before and after transnasal-transsphenoidal tumor removal in a comparative study design. Methods: A series of 100 patients with PitNet were analyzed. We compared demographic data (age, sex, BMI), comorbidities, tumor volume and neuropathological findings between patients who presented with hemorrhagic PitNet (H-PitNet) and those with non-hemorrhagic PitNet (NH-PitNet). Furthermore, we compared the axis-specific hormonal status between both groups at admission and after microsurgical tumor removal and analyzed the overall endocrine outcome in both groups. Results: A total of 22 patients presented with hemorrhagic PitNet. There were no differences in age, sex, BMI, tumor volume, smoking status or diabetes status between patients with H-PitNet and those with NH-PitNet. H-PitNet was strongly associated with necrotic areas (*p* < 0.0001). Corticotropic PitNet was slightly overrepresented in H-PitNet (*p* = 0.04). Arterial hypertension was more frequent in patients with H-PitNet (*p* = 0.009). The presence of hypopituitarism in each axis at admission and after surgery was comparable between the two groups. In total, there were fewer recovering axes (*p* = 0.03) and more axes with persistent deficiency (*p* = 0.01) in the H-PitNet group after surgery. Conclusions: H-PitNet is associated with previous vascular event (ischemia or hemorrhage) and the presence of arterial hypertension and results in worse endocrine outcome. Early surgery should be considered in patients with PitNet and arterial hypertension.

## Simple Summary

This study analyzed 100 patients with pituitary neuroendocrine tumors (PitNets) to identify factors associated with hemorrhage and examine hormonal outcomes before and after surgery. This research found that hemorrhagic PitNet was linked to necrotic areas within tumors and a higher frequency of arterial hypertension. Despite similar demographics and tumor characteristics between hemorrhagic and non-hemorrhagic PitNet patients, those with H-PitNet had worse endocrine outcomes after surgery, with more persistent hormone deficiencies. The findings suggest that H-PitNet is associated with prior vascular events and that early surgical intervention should be considered for patients with PitNet and arterial hypertension to improve endocrine recovery.

## 1. Introduction

Pituitary neuroendocrine tumors (PitNets, formerly called pituitary adenomas) are particularly susceptible to intratumoral hemorrhages. Hemorrhages occur 5.4 times more often in PitNets than in other brain tumors [1]. Such hemorrhages can have a subclinical course or may lead to clinical pituitary apoplexy, which was defined by Brougham et al. as hemorrhagic or necrotic degenerated PitNets associated with acute onset of neurological symptoms, including visual loss, ophthalmoplegia and decreased consciousness, which can be a life-threatening condition [2]. It occurs in up to 20% of patients with PitNet and is associated with a mortality rate ranging from 1 to 5% [3].

Over the years, a number of precipitating factors have been identified, including head trauma, surgery, hormone therapy, bromocriptine therapy, pregnancy, radiotherapy, endocrine dynamic tests, anticoagulation, and others [3,4,5]. Patient- and tumor-related risk factors, however, have received less attention. Furthermore, there is no consensus regarding the optimal therapeutic management [3]. While most investigators recommend expeditious surgery when neurologic deficits are present [6], a conservative approach has been advocated as well [7].

The goal of this study was to provide data on factors associated with an increased risk for hemorrhage in patients with PitNet. We therefore aimed to (1) identify patient- and tumor-related risk factors for intratumoral hemorrhage in PitNet and (2) review and compare the endocrine outcomes of patients undergoing microsurgical resection for H-PitNet and non-hemorrhagic PitNet (NH-PitNet).

## 2. Methods

### 2.1. Study Design

**Figure 1:**
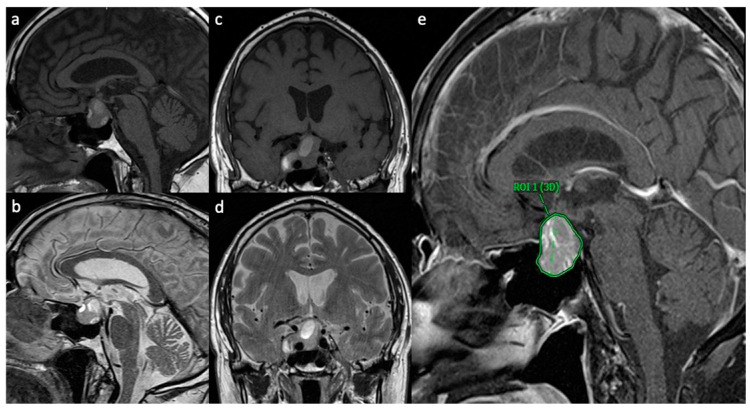
**Representative MRI scans of H-PitNet with multiple hemorrhagic lesions.** (**a**) Sagittal T1W MRI showing T1 hyperintense hemorrhagic lesions in a PitNet; (**b**) sagittal MRI showing T2 hyperintense lesions in a PitNet. The same lesions are displayed in the coronal view in (**c**) (T1W) und (**d**) (T2W). A sagittal view of contrast-enhanced T1W MRI is displayed in (**e**), showing an inhomogeneous contrast enhancement on the PitNet, which is marked as a 3D region of interest (ROI) for volumetry.

The medical records of a consecutive series of 100 patients who presented with a PitNet at the Department of Neurosurgery of Hannover Medical School between 2017 and 2022 were analyzed retrospectively. The patients were divided into two groups (hemorrhagic PitNet: H-PitNet; and non-hemorrhagic PitNet: NH-PitNet) based on the preoperative MRI findings, neuropathologic findings and clinical presentation. Hemorrhages were identified on non-enhanced T1- and T2-weighted MRI, as described by Wang et al. (see Figure 1) [8].

To identify possible factors associated with hemorrhage, we compared age, sex, BMI, tumor volume, neuropathological findings, smoking status, the presence of arterial hypertension (when previously diagnosed and treated with antihypertensive agents) and of diabetes mellitus between the two groups. The tumor volume was measured using semiautomated 3D-ROI-based volumetry (software: Visage Imaging Inc. ©, V 7.1.18, 2020, San Diego, CA, USA; see Figure 1), as described previously [9].

### 2.2. Endocrine Status

To assess possible differences in endocrine status at admission or after surgery, we performed a group comparison for each hormonal axis pre- and postoperatively. To evaluate the overall outcome in a cross-axis way, we analyzed the number of axes showing recovery or persistent hypopituitarism after surgery.

The preoperative pituitary hormonal status was determined by laboratory examinations, including serum levels of prolactin, luteinizing hormone (LH), follicle-stimulating hormone (FSH), testosterone, estrogen, adrenocorticotropic hormone (ACTH), cortisol, thyroid-stimulating hormone (TSH), thyroxine (T4), triiodothyronine (T3), growth hormone, insulin-like growth factor 1 (IGF1), and insulin-like growth factor-binding protein 3 (IGFBP3).

A re-evaluation of the hormonal status was performed 6 weeks after surgery with the aforementioned parameters and a corticotropin-releasing hormone test (CRH test). Hypopituitarism was diagnosed based on laboratory results according to our local laboratory reference values.

### 2.3. Data Analysis

Age, BMI and tumor volume were expressed as mean ± SD and were analyzed using the Mann–Whitney test. Ki-67 and the number of axes recovering or showing persistent deficiency were expressed as mean ± SD and were analyzed by Student’s t test and Welch’s t test when variances were different between groups. Categorical variables (sex, arterial hypertension, diabetes mellitus and smoking status, visual impairment, extent of resection, necrosis, histological subtype, and AE1/3) were compared with Fisher’s exact test. Parameters showing a significant difference between groups were included in a multiple logistic regression model. The presence (or absence) of hypopituitarism on each axis was analyzed by Fisher’s exact test.

Multivariate analysis was performed with an online statistical tool (https://statisty.app/logistic-regression-calculator, accessed on 25th of May 2024). All other statistical analyses and graphs were generated with GraphPad Prism (GraphPad Prism 9.3.0 (345) Macintosh Version by Software MacKiev © 1994–2021 GraphPad Software, LLC, San Diego, CA, USA). Statistical significance was defined as *p* < 0.05.

## 3. Results

### 3.1. Cohort Description

H-PitNet group: A total of 22 patients, including 16 men and 6 women aged 35 to 83, were identified as having a H-PitNet. All patients presented with a macroadenoma. The laboratory results indicated a prolactinoma in one patient. Only two patients were receiving anticoagulant therapy. Other possibly precipitating factors were found in four patients: two patients had previous cardiac surgery, one patient had a traumatic brain injury, and the patient with prolactinoma was receiving bromocriptine therapy. Most patients (N = 16; 73%) presented with the clinical picture of a pituitary apoplexy with acute onset symptoms, including severe headache and visual impairment. One patient presented with acute hydrocephalus and decreased consciousness. In two patients, a conservative approach (wait and scan) was prioritized. The remaining patients underwent microsurgical transnasal-transsphenoidal resection. The median follow-up time was 25 months.

NH-PitNet group: The NH-PitNet group consisted of 78 patients. There were 43 men and 35 women aged 20 to 86 years. A total of four microadenomas were found in this group. Functioning PitNets were found in 10 patients (two prolactinomas, one ACTH-secreting adenoma, one FSH-secreting adenoma and six growth-hormone-secreting adenomas). One patient was under anticoagulant therapy. All subjects in the NH-PitNet group underwent microsurgical transnasal-transsphenoidal resection, except one. The median follow-up time was 40 months.

### 3.2. Histological Findings

**Figure 2:**
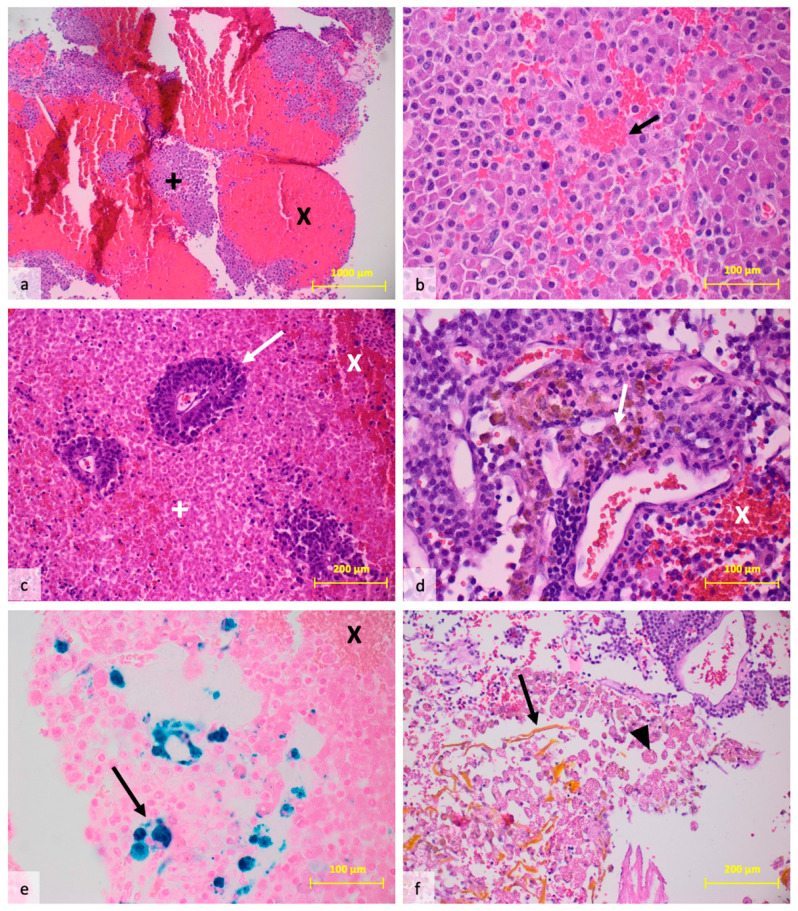
**Microscopic images of various pathological findings in H-PitNet.** (**a**) Massive, solid, fresh hemorrhage (X) in an adenoma (+), which consequently dissociates the tissue (HE staining, 4× resolution); (**b**) diffuse hemorrhages in the sense of microbleedings (arrow) within a structurally still intact adenoma (HE staining, 40× resolution); (**c**) extensive geographic necrosis (+), within small vital islands of an adenoma (arrow) and fresh hemorrhages (X) are still detectable (HE staining, 20× resolution); (**d**) older remnants of brownish hemorrhage (arrow) as well as a fresh bleeding component (X) within an adenoma (HE staining, 40× resolution); (**e**) the older remnants of hemorrhage demark themselves as blue-stained hemosiderin (arrow) with an additional fresh bleeding component (X) (Prussian blue staining, 40× resolution); (**f**) detection of considerably older hematoidin (arrow) in regions with resorbing foamy macrophages (triangle) (HE staining, 20× resolution).

A microscopic neuropathological evaluation of hemorrhagic PitNETs was available in 19 patients. Seven PitNETs presented as a gonadotropic subtype (37%), and five PitNETs as a corticotropic subtype (27%). Five tumors were endocrine-inactive (27%). Immunohistochemical characterization was not possible in two patients. Solid, extensive, fresh hemorrhages were found in 18/19 of the tumors (95%). In one PitNET, only diffuse, sparse hemorrhage could be detected (5%). In 10 of the 19 PitNETs, an older bleeding component in the form of hemosiderin was found in addition to fresh hemorrhage (53%). In 2 of the 10 patients, older hematoidin components were demonstrated. All 10 patients with an older bleeding component exhibited fresh, solid, extensive hemorrhages. A total of 10 PitNETs demonstrated necrosis (53%). In 7/10 of these patients, there was an older bleeding component (70%), and three patients with tumors with an older bleeding component had no necrosis (30%). The necroses consistently showed shadows of deceased tumor cells. Signs of resorption in the form of foamy macrophages were present in 7/19 of the PitNETs (37%). Resorption was found only in tumors with necrosis. In all patients, the intratumoral blood vessels showed no pattern of microangiopathy. The Ki-67 proliferation rate was between 1 and 4%, with a mean of 2%. Synaptophysin was consistently expressed in 18 tumors. AE1/3 expression was analyzed in 15 patients and was positive in 10 patients (66%). The neuropathological findings in H-PitNet are summarized in Table 1. Exemplary images are displayed in Figure 2.

A total of 76 NH-PitNets were neuropathologically examined. Gonadotropic and corticotropic subtypes were found in 38 and 7 patients, respectively. Necrosis was found in two patients (3%). The Ki-67 proliferation rate ranged from 1 to 15%. Synaptophysin was detected in all tumors. AE1/3 was expressed in 41 patients.

### 3.3. Comparative Analysis of Factors Possibly Associated with Hemorrhage in PitNets

**Figure 3:**
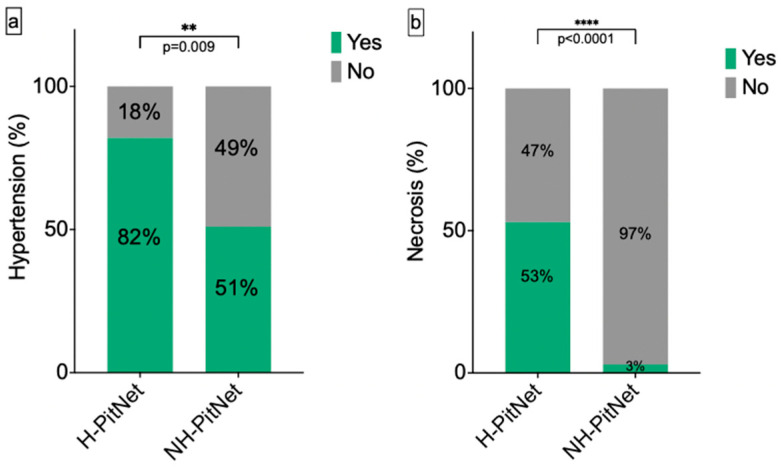
**Frequency of arterial hypertension and tumor necrosis in H-PitNet and NH-PitNet.** (**a**) Arterial hypertension was significantly more frequent in H-PitNet (82% vs. 51%; **: *p* = 0.009). (**b**) Tumor necrosis was present in the majority of H-PitNet but only in a few cases of NH-PitNet (53% vs. 3%, ****: *p* < 0.0001).

There was no significant difference in age (H-PitNet: 59 ± 14.83 years; NH-PitNet: 60 ± 14.09 years; *p* = 0.66), BMI (H-PitNet: 29 ± 5.75 kg/m^2^; NH-PitNet: 29 ± 6.19 kg/m^2^; *p* = 0.33) or sex (*p* = 0.31) between the two groups. There was also no difference in smoking status (*p* = 0.17) or diabetes mellitus (*p* = 0.47) between groups. The tumor volume was similar in both groups (H-PitNet: 8771 ± 10,339 mm^3^; NH-PitNet: 8748 ± 12,452 mm^3^; *p* = 0.86). Arterial hypertension was more frequent in the H-PitNet group (82% vs. 51%; *p* = 0.009; Figure 3a).

Necrosis was strongly associated with hemorrhagic PitNet (53% vs. 3%; *p* < 0.0001; Figure 3b). Corticotropic PitNet was significantly more frequent in hemorrhagic PitNet (29% vs. 9%; *p* = 0.04). There was no significant difference regarding the KI-67 proliferation rate (H-PitNet: 2 ± 1.06%; NH-PitNet: 2.18 ± 1.99%; *p* = 0.61), AE1/3 expression (*p* = 0.77) or gonadotropic subtype (*p* = 0.59). An overview of the results is shown in Table 2.

Multiple logistic regression analysis of arterial hypertension and the corticotropic subtype confirmed that arterial hypertension was independently associated with H-PitNet (*p* = 0.02, OR = 3.99, 95% CI: 1.23–13.01).

### 3.4. Extent of Resection

A total of 20 patients in the H-PitNet group underwent microsurgical transnasal-transsphenoidal resection and 77 patients underwent surgery in the NH-PitNet group. Gross total resection could be achieved in 15 patients in the H-PitNet group and in 59 patients in the NH-PitNet group (*p* > 0.99). In a 49-year-old woman, hemorrhage led to a subarachnoid hemorrhage and decreased consciousness at admission; she died after surgery.

### 3.5. Visual Impairment and Diplopia

On admission, decreases in visual acuity and diplopia were more frequent in patients with H-PitNet (visual loss: 64% vs. 24%, *p* = 0.001; diplopia: 41% vs. 6%, *p* = 0.0003). There was no difference in the frequency of visual field deficits (*p* = 0.45). The frequency of visual impairment and diplopia is summarized in Table 3.

### 3.6. Pituitary Hormonal Status

Only patients with nonfunctioning adenoma who underwent microsurgical transnasal-transsphenoidal surgery were included in this analysis. For each axis, only patients with complete laboratory sets were considered. One patient who died postoperatively was excluded from this analysis due to the absence of available laboratory sets. The hormonal status of patients with H-PitNet is summarized in Table 4.

#### 3.6.1. Preoperative Hormonal Status

Corticotropic axis: The preoperative corticotropic hormonal status was available for 15 and for 64 patients in the H-PitNet and the NH-PitNet groups, respectively. Corticotropic deficiency was found in five patients in the H-PitNet group and in nine patients in the NH-PitNet group, but the difference was not statistically significant (H-PitNet: 33%, NH-PitNet: 14%, *p* = 0.13).

Thyrotropic axis: The thyrotropic axis was preoperatively assessed in 15 patients in the H-PitNet group and in 61 patients in the NH-PitNet group. There was no difference in thyrotropic insufficiency between the groups (H-PitNet: 80%, NH-PitNet: 52%, *p* = 0.08).

Gonadotropic axis: The preoperative gonadotropic hormonal status was analyzed in 14 subjects in the H-PitNet group and in 60 in the NH-PitNet group. The group comparison did not reveal differences in gonadotropic insufficiency (H-PitNet: 71%, NH-PitNet: 62%, *p* = 0.55).

Somatotropic axis: Preoperative gonadotropic hormonal status was assessed in 14 patients in the H-PitNet group and in 60 patients in the NH-PitNet group. There was no difference in somatotropic insufficiency between the two groups (H-PitNet: 21%, NH-PitNet: 22%, *p* > 0.99).

#### 3.6.2. Postoperative Hormonal Status

Corticotropic axis: The postoperative corticotropic hormonal status was available for 18 patients in the H-PitNet group and for 63 in the NH-PitNet group. Corticotropic deficiency was found in 9 and 20 patients in the H-PitNet and NH-PitNet groups, respectively (H-PitNet: 50%, NH-PitNet: 32%, *p* = 0.17).

Thyrotropic axis: Postoperative thyrotropic hormonal status was assessed in 17 and 58 subjects in the H-PitNet and NH-PitNet groups, respectively. The statistical analysis revealed no differences in thyrotropic deficiency between the two groups (H-PitNet: 53%, NH-PitNet: 33%, *p* = 0.16).

Gonadotropic axis: Gonadotropic hormonal status was postoperatively determined in 18 patients in the H-PitNet group and in 63 patients in the NH-PitNet group. Gonadotropic insufficiency was found in 12 and 30 subjects in the H-PitNet and NH-PitNet groups, respectively (H-PitNet: 67%, NH-PitNet: 48%, *p* = 0.19).

Somatotropic axis: Postoperative somatotropic hormonal status was evaluated in 18 patients in the H-PitNet group and in 62 patients in the NH-PitNet group. Somatotropic deficiency was detected in five subjects in the H-PitNet group and in 11 in the NH-PitNet group (H-PitNet: 28%, NH-PitNet: 18%, *p* = 0.34).

#### 3.6.3. Overall Endocrine Outcome

**Figure 4:**
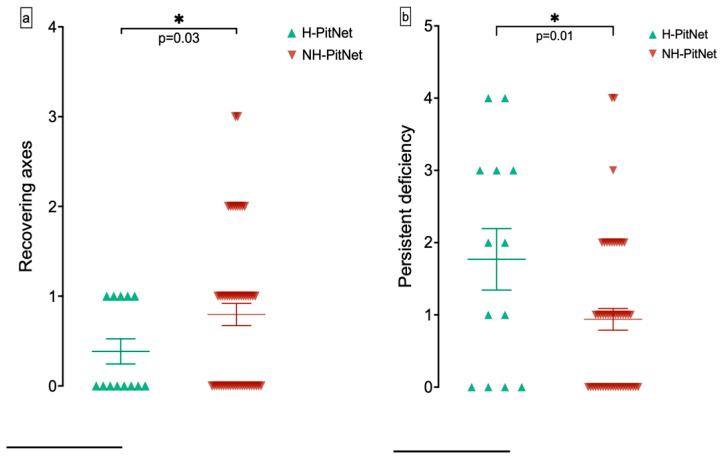
**Number of endocrinological axes recovering from hypopituitarism or showing persistent hypopituitarism after surgery.** (**a**) The number of axes recovering from hypopituitarism was higher in NH-PitNet (*: *p* = 0.03). (**b**) The number of axes with persistent hypopituitarism was higher in H-PitNet (*: *p* = 0.01). The triangles represent individual values, while the bars indicate the mean ± SD.

A total of 13 patients with H-PitNet presented with hypopituitarism on at least one axis, where there were 51 patients with hypopituitarism in the NH-PitNet group (H-PitNet: 72%, NH-PitNet: 81%; *p* = 0.51). Moreover, we examined the number of axes that recovered from hypopituitarism or showed persistent hypopituitarism after microsurgical resection. While the number of recovering axes was significantly lower in patients with H-PitNet (H-PitNet: 0.43 ± 0.51, NH-PitNet: 0.80 ± 0.86, *p* = 0.03), the number of axes with persistent hypopituitarism was greater in these patients (H-PitNet: 1.71 ± 1.49; NH-PitNet: 0.94 ± 1.05; *p* = 0.01). These results are displayed in Figure 4.

## 4. Discussion

The identification of factors associated with hemorrhage into a PitNet adenoma has been the subject of several studies in the past. In our study, we focused on the potential value of patient- and tumor-related parameters, including demographic data, comorbidities, tumor volume and neuropathological findings.

Our data revealed a strong association between hemorrhage and tumor necrosis. Necrosis in PitNet has previously been described after ischemic infarction and/or intratumoral hemorrhage [10,11,12]. In our cohort, 53% of the patients with H-PitNet presented with necrosis that was partially older than the acute hemorrhage. This finding suggests that the new hemorrhage occurred subsequent to a prior vascular event (hemorrhagic or ischemic infarction). This is even more remarkable in so far as signs of older bleeding were found in 70% of patients. Ebersold et al. reported thrombosis in the sinusoids and suggested that vascular stasis might be the underlying factor leading to ischemic necrosis and subsequently to hemorrhage [13]. Our data indicate that there might be an underestimated risk of (re)bleeding in patients with hemorrhagic or ischemic PitNet.

The immunohistochemical analyses revealed a slight overrepresentation of corticotropic PitNet in patients who presented with hemorrhage. However, this difference was not enough to predict hemorrhage in the multivariate analysis. In their series of 81 patients with H-PitNet, Lui et al. reported corticotropic tumors in only 6% of the patients. They found that the null cell PitNet was associated with H-PitNet [14].

Our results show that arterial hypertension is associated with hemorrhage in PitNet, which is in line with previous findings [15]. It has been shown that the vascular density is reduced in PitNets and that abnormal vessels are present [16,17,18]. In particular, in this context, it is plausible that arterial hypertension would increase the risk of bleeding. It needs to be mentioned that some studies did not find arterial hypertension to be associated with hemorrhage within PiNets [8,14,19]. This difference between studies may be explained both by selection and real bias, i.e., inherent problems of retrospective studies.

The presence of diabetes mellitus and smoking status were not associated with intratumoral hemorrhage in our cohort. Möller-Goede et al. and Liu et al. also investigated the predictive value of diabetes mellitus for hemorrhagic pituitary adenoma and found no correlation [14,19]. Moreover, our data suggest that sex, age and BMI are not associated with hemorrhage into PitNets. Particularly, in that regard, previously published data are very heterogeneous. Although Möller-Goede et al. and Araujo-Castro et al. reported that male sex was an independent factor associated with intratumoral bleeding, Sarwar et al. noted that female sex was associated with bleeding [19,20,21]. In their study, Wakai et al. suggested that hemorrhages within PitNets are not associated with sex but, instead, are associated with age [1]. Nonetheless, other investigators agree that neither age nor sex are correlated with hemorrhages within PitNets [8,14,22]. In contrast to our results, Araujo-Castro et al. reported that obesity is associated with pituitary apoplexy [21]. Again, such contradictory results might be due to the selected cohorts and their constitution regarding functioning and nonfunctioning pituitary adenomas. For example, our cohort consisted almost entirely of nonfunctioning adenomas (with the exception of one prolactinoma), while Sarwar et al. included only prolactinomas in their study [20].

Tumor size has been postulated to predict hemorrhage in PitNet according to some studies [8,19,20]. Given that calculating the tumor volume in PitNet is challenging due to the irregular shape of the tumor, some studies used only the diameter of the tumor as a surrogate for the volume. In this study, we performed more reliable tumor volumetry in both H-PitNets and NH-PitNets and observed that the tumor volume was similar in both groups. According to our observations, tumor size may not be a primary risk factor for PitNets.

In our cohort, H-PitNet did not affect adenohypophyseal function more often than NH-PitNet did at the axis level. But there were fewer axes that recovered after surgery in the H-PitNet group, while the number of axes with persistent hypopituitarism was greater. The underlying mechanisms of hypopituitarism include several findings, such as tumor mass effects, resulting in compression of the pituitary and/or its stalk, and impaired blood circulation in the healthy pituitary tissue [23]. It appears that the pituitary axes are generally not equally affected by hypopituitarism in NH-PitNet. While the somatotropic and gonadotropic axes are often affected, thyrotropic and corticotropic deficiencies are less common [23]. The lack of a significant difference between groups in hypopituitarism on any axis suggests that hemorrhage into pituitary adenomas does not affect the sensitivity of the different pituitary cell types considering functional loss. The endocrine outcome in H-PitNets has often been reported to be generally satisfactory [15,24,25,26]. Remarkably, however, compared to NH-PitNet, the overall endocrine outcome was worse in H-PitNet in our cohort, even though no differences were found at the axis level. These findings are consistent with those of Wang et al., who reported no differences in endocrine outcomes between H-PitNet and NH-PitNet at the axis level [8]. Regarding the overall endocrine outcome, Möller-Goede et al. and Araujo-Castro et al. also noted worse outcomes in patients suffering from pituitary apoplexy [19,21]. Our data, more specifically, show that the pituitary axes recover less often and show persistent deficiency more often in patients with H-PitNets. This might be due to the acute mass effect of tumor bleeding, causing cell death in the pituitary. In that regard, the findings of Zayour and colleagues, who reported a marked elevation in intrasellar pressure in patients with pituitary apoplexy, need to be mentioned [27]. Even though it has decreased in recent years, mortality in pituitary apoplexy patients should not be neglected [3]. We also experienced one case of death in our cohort.

In summary, H-PitNet is associated with worse outcomes and therefore deserves particular attention.

Limitations of our study are its retrospective design and the fact that endocrinological studies were not available for every patient, especially for patients who underwent emergency surgery. Patients have varying sets of laboratory data available, which makes extensive comparison challenging. A subgroup analysis of subclinical vs. symptomatic hemorrhages would have been of interest but was not possible since most patients had an acute onset of symptoms.

## 5. Conclusions

Our study provides a unique point of view on H-PitNets by emphasizing the associated risk of (re)bleeding after a previous vascular event. H-PitNets are associated with worse outcomes than NH-PitNets. In patients who present with radiologic signs of hemorrhage or necrosis, the risk of (re)bleeding and apoplexy should be considered when making treatment decisions. Surgical treatment might be advocated especially for patients with PitNet and arterial hypertension.

## Figures and Tables

**Table 1:** Histological findings in 19 patients with H-PitNet.

	Subtype	Fresh Hemorrhage	Hemosiderin/Hematoidin	Necrosis	Ki-67 (%)
1	Null cell	+	+	+	1
2	Corticotropic	+	−	+	3
3	Gonadotropic	+	+	+	1
4	n.a.	+	+	+	n.a.
5	Gonadotropic	+	+	+	n.a.
6	Null cell	+	+	−	3
7	Gonadotropic	+	+	−	1
8	Null cell	+	−	−	3
9	Corticotropic	+	+	+	3
10	Gonadotropic	+	+	+	2
11	Gonadotropic	+	−	+	3
12	Corticotropic	+	+	−	1
13	Gonadotropic	+	−	+	1
14	Null cell	+	−	−	4
15	n.a.	+	−	−	3
16	Corticotropic	+	−	−	1
17	Gonadotropic	+	+	+	1
18	Null cell	+	−	−	1
19	Corticotropic	+	−	−	2

**Table 2:** Comparative analysis of factors possibly associated with hemorrhage in PitNets.

	H-PitNet (N = 22)	NH-PitNet (N = 78)	*p* Value
Age (years)	59 ± 14.83	60 ± 14.09	0.66
Sex (M/F)	16/6	43/35	0.15
BMI (kg/m^2^)	29 ± 5.75	29 ± 6.19	0.33
Tumor volume (mm^3^)	8771 ± 10,339	8748 ± 12,452	0.86
Arterial hypertension (yes/no)	18/4	40/38	0.009
Diabetes mellitus	4/18	9/69	0.47
Smoker (yes/no)	4/18	21/57	0.58
Necrosis (yes/no)	10/9	2/74	<0.0001
AE1/3 (positive/negative)	10/5	41/26	0.77
Ki-67	2 ± 1.06	2.18 ± 1.99	0.61
Histologic subtype			
Gonadotropic (yes/no)	7/10	38/36	0.59
Corticotropic (yes/no)	5/12	7/67	0.04

**Table 3:** Frequency of visual impairment and diplopia in patients with H-PitNet and NH-PitNet.

	H-PitNet	NH-PitNet	*p* Value
Visual loss (yes/no)	14/8	19/59	0.001
Diplopia (yes/no)	9/13	5/73	0.0003
Visual field deficits (yes/no)	6/16	29/49	0.45

**Table 4:** Endocrine status in patients 18 patients with H-PitNet.

	Age	Sex	Preoperative Hypopituitarism				Postoperative Hypopituitarism			
			Corticotropic	Thyrotropic	Gonadotropic	Somatotropic	Corticotropic	Thyrotropic	Gonadotropic	Somatotropic
1	68	M	Yes	Yes	Yes	Yes	Yes	Yes	Yes	Yes
2	56	M	Yes	Yes	Yes	No	Yes	Yes	Yes	Yes
3	35	F	n.a.	n.a.	n.a.	n.a.	No	No	No	No
4	56	M	No	Yes	Yes	Yes	Yes	No	Yes	Yes
5	45	M	No	Yes	No	No	Yes	No	No	No
6	61	M	No	No	No	No	No	Yes	Yes	No
7	79	M	No	No	No	No	No	No	No	No
8	67	M	n.a.	yes	n.a	n.a.	No	Yes	Yes	Yes
9	52	M	No	n.a.	n.a.	n.a.	No	n.a.	No	No
10	54	F	No	Yes	No	No	No	No	No	No
11	83	M	No	Yes	Yes	No	Yes	No	Yes	No
12	50	M	Yes	Yes	Yes	Yes	Yes	Yes	Yes	Yes
13	67	M	Yes	Yes	Yes	No	Yes	Yes	Yes	No
14	44	F	n.a	n.a	n.a.	n.a.	Yes	Yes	Yes	No
15	42	M	No	Yes	Yes	No	No	No	Yes	No
16	35	M	No	Yes	Yes	No	No	Yes	Yes	No
17	48	F	No	No	Yes	No	No	No	No	No
18	72	F	Yes	Yes	Yes	No	Yes	Yes	Yes	No

## Data Availability

The data that support the findings of this study are available from the corresponding author, HFH, upon reasonable request.
